# Management with ketorolac or corticosteroids for subacromial impingement syndrome: Results from a randomised controlled trial

**DOI:** 10.1002/jeo2.70648

**Published:** 2026-01-26

**Authors:** Omid Salkhori, Mahdi Sahebi, Mohammad Reza Guity, Saman Ghiasi Nezhad, Nima Bagheri

**Affiliations:** ^1^ Joint Reconstruction Research Center Tehran University of Medical Sciences Tehran Iran; ^2^ Department of Orthopedic Surgery, Imam Khomeini Hospital Complex Tehran University of Medical Sciences Tehran Iran; ^3^ Students’ Scientific Research Center (SSRC) Tehran University of Medical Sciences Tehran Iran

**Keywords:** corticosteroid, functional outcomes, ketorolac, range of motion, subacromial impingement syndrome

## Abstract

**Purpose:**

Subacromial impingement syndrome (SAIS) causes shoulder pain and limitations. While corticosteroid injections are common, concerns about side effects lead to exploring alternatives like ketorolac. This trial compared the short‐term effectiveness of subacromial ketorolac versus corticosteroids in SAIS patients.

**Methods:**

This double‐blind, randomised controlled trial enrolled 120 patients with clinically diagnosed SAIS. Participants were randomly assigned to receive a single ultrasound‐guided subacromial injection of either ketorolac (30 mg) or methylprednisolone acetate (40 mg). The primary outcome was change in active shoulder forward flexion at 3 months. Secondary outcomes included other shoulder range‐of‐motion measures, shoulder abduction strength, the simple shoulder test (SST) and the Oxford shoulder score (OSS). Between‐group comparisons were performed using baseline‐adjusted analyses of covariance, with a noninferiority margin of −10° prespecified for the primary outcome.

**Results:**

A total of 118 patients completed the 3‐month follow‐up and were included in the final analysis (two dropouts). Both groups demonstrated statistically significant improvements from baseline in shoulder range of motion, strength and patient‐reported outcomes (*p* < 0.001). The adjusted mean difference in forward flexion between the ketorolac and corticosteroid groups was 0.07° (95% CI, −4.40° to 4.26°), meeting the prespecified criterion for noninferiority. No clinically meaningful between‐group differences were observed for secondary outcomes. No injection‐related adverse events were reported during follow‐up.

**Conclusion:**

Subacromial ketorolac injection produced short‐term improvements comparable to those of corticosteroid injection in patients with SAIS. Ketorolac was non‐inferior to corticosteroids for shoulder forward flexion at 3 months. Longer‐term studies are needed to confirm these findings.

**Level of Evidence:**

Level I.

## Graphical abstract

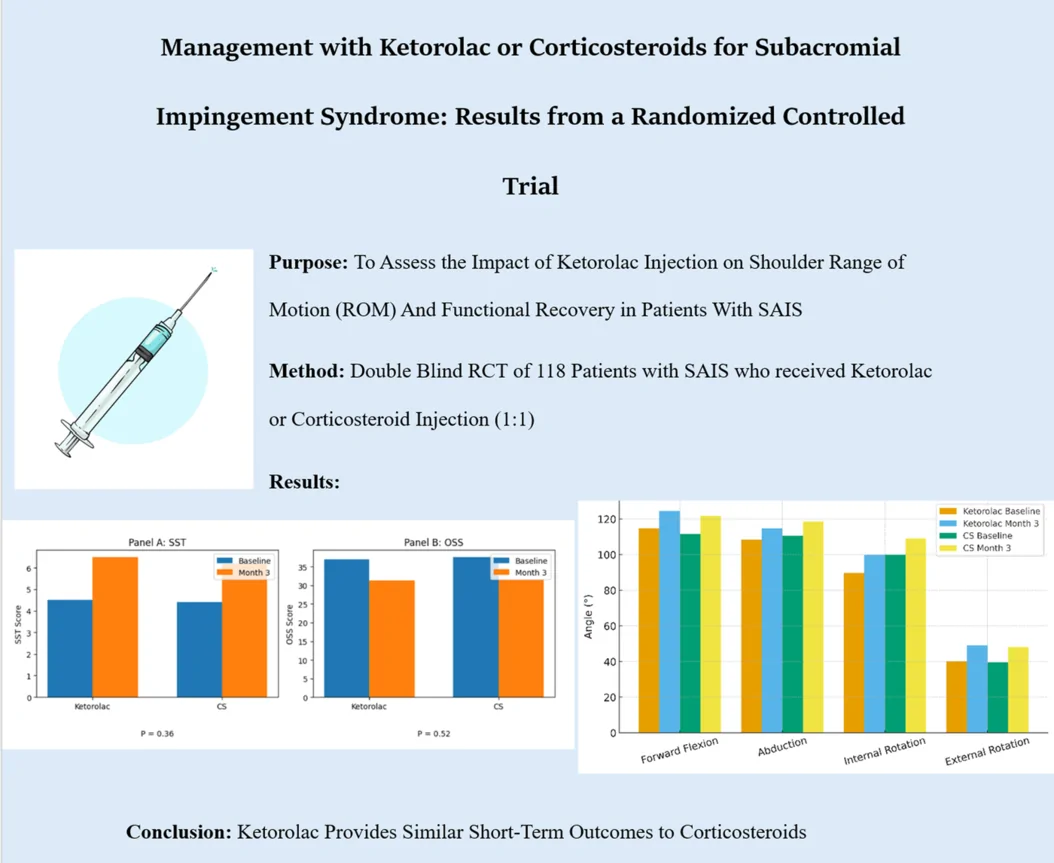

**Abbreviations**
CS: corticosteroid
IRCT: Iranian Registry of Clinical Trials
MRC: Medical Research Council (Manual Muscle Testing Scale)
MRI: magnetic resonance imaging
NSAID: nonsteroidal anti‐inflammatory drug
OSS: Oxford shoulder score
RCT: randomised controlled trial
ROM: range of motion
SAIS: subacromial impingement syndrome
SD: standard deviation
SPADI: Shoulder pain and disability index
SPSS: Statistical Package for the Social Sciences
SST: simple shoulder test
TUMS: Tehran University of Medical Sciences
UCLA: University of California at Los Angeles (Shoulder Score)
VAS: visual analog scale

## INTRODUCTION

Subacromial impingement syndrome (SAIS) is the most common manifestation of shoulder disorder, making up about 44% to 65% of all shoulder pain complaints [5, 23]. SAIS encompasses various subacromial space conditions, such as rotator cuff tears, calcific tendinitis, biceps tendinopathy, rotator cuff tendinosis and subacromial bursitis [15]. Shoulder impingement occurs due to inflammation and degeneration of structures in the subacromial space [16]. Both intrinsic and extrinsic mechanisms have been suggested for this syndrome; however, it remains unclear whether rotator cuff tendon damage leads to impingement (intrinsic mechanism) or if impingement itself causes the tendon damage (extrinsic mechanism) [5]. Several external factors can contribute to rotator cuff disease and shoulder pain, such as heavy physical workload, vibration exposure, injury, smoking, infection, fluoroquinolone use and genetic predispositions [7, 9, 25].

Traditionally, acromioplasty with bursectomy has been used for patients unresponsive to conservative care [5]. In recent years, experts questioned the need for surgery. Advances in disease understanding have led studies to challenge decompression procedures and the ‘impingement’ theory, suggesting that placebo effects, rest and physiotherapy (e.g., shoulder stretching, serratus anterior strengthening and upper trapezius activation reduction) are effective treatments. Current literature indicates that conservative management effectively resolves shoulder impingement syndrome in most cases, especially for individuals experiencing mild to moderate symptoms [13].

Corticosteroid (CS) injections are frequently used to treat SAIS that hasn't responded to conservative therapies [2, 28]. Although their exact mechanism isn't completely understood, they are thought to reduce inflammation in cases of bursitis and tendonitis [28]. However, these injections are limited because of serious side effects such as tendon rupture, subcutaneous atrophy, cartilage changes and systemic effects like osteoporosis [19, 22]. Since CSs mainly work through anti‐inflammatory effects, nonsteroidal anti‐inflammatory drug (NSAID) injections could reduce subacromial inflammation similarly. Current literature suggests emerging alternatives such as NSAIDs and Platelet Rich Plasma injections may alleviate pain and improve function in shoulder disorders [4, 8, 12, 24]. Ketorolac, an NSAID, inhibits prostaglandin synthesis and relieves moderate‐to‐severe pain [22]. This study aims to compare the clinical effectiveness of subacromial Ketorolac injection with CS injection in patients with SAIS. The primary aim is to assess improvements in shoulder forward flexion at 3‐month follow‐up. Secondary aims include evaluating changes in shoulder range of motion (ROM), abduction strength and patient‐reported outcomes, specifically the simple shoulder test (SST) and the Oxford shoulder score (OSS). We hypothesise that Ketorolac injection will provide pain relief and functional improvement comparable to CS injection, resulting in similar gains in shoulder mobility and patient‐reported outcomes, while offering a potentially safer alternative by avoiding CS‐related adverse effects.

## MATERIALS AND METHODS

### Study design and ethical approval

A double‐blind randomised controlled trial study was conducted on patients diagnosed with SAIS at the orthopaedic clinic of Imam Khomeini Hospital Complex, a nationwide hospital in Tehran, Iran, affiliated with the Tehran University of Medical Sciences. The ethical approval was obtained by the institutional review board of the Tehran University of Medical Sciences (IR.TUMS.IKHC.REC.1401.315) before enrolling the patients, and written consent was obtained from all the participants. The aims of the study, confidentiality, and freedom of participation were explained to all patients before enrolling. This trial was registered in the Iranian registry of clinical trials (irct.behdasht.gov.ir, registration code: IRCT20230625058584N1).

### Enrolment of participants

The inclusion criteria were as follows: (a) aged between 25 and 80 years, (b) both sexes, (c) SAIS diagnosis with clinical signs (e.g., pain in the shoulder and restriction of active motion), (d) no contraindications for shoulder injection and (e) the ability to give informed consent. The patients were excluded if they had: (a) previous history of shoulder injection (within the last 3 months) (b) any history of shoulder surgery, (c) presence of rotator cuff tear on ultrasonography or magnetic resonance imaging (MRI), (d) complete rotator cuff tear or other pathologies of shoulder (glenohumeral arthrosis, calcific tendinitis, shoulder instability and acromioclavicular joint pathology), (e) active tumour or haematological malignancies, (f) coagulation disorders or anticoagulant use and and (g) infection.

### Sample size calculation

The determination of the minimum requisite sample size for the two groups was performed using the G*Power 3.1.9.2 software (http://www.gpower.hhu.de/en.html, accessed 1 January 2016), before participant enrolment. Based on a calculated effect size of 0.5, an *α* level of 0.05, a power of 80% and an allocation ratio of 1:1, the a priori power analysis determined a total sample size of at least 102 participants.

### Randomisation and allocation concealment

Participants were randomly assigned in a 1:1 ratio to receive either ketorolac or CS (methylprednisolone) injection. Randomisation was performed using a computer‐generated random sequence by an independent researcher not involved in patient recruitment or assessment. Allocation concealment was ensured using sequentially numbered, opaque, sealed envelopes, which were opened only at the time of injection.

### Blinding

This was a double‐blind study. Participants, the outcome assessor, and the statistician were blinded to group allocation. Injections were prepared by a nurse not involved in outcome assessment. Both injections were administered in identical syringes with the same volume and appearance. The treating orthopaedic specialist performing the injection was not involved in outcome assessment and had no access to follow‐up data.

### Ketorolac and CS injection

All injections were performed by a single experienced orthopaedic specialist under ultrasound guidance using a standardised posterolateral approach. Participants of both groups received 2 mL injections. Patients of the Ketorolac group were injected with a mixture of 1 mL of ketorolac (30 mg/mL) and 1 mL of lidocaine hydrochloride 2%. CS group patients received an injection of 1 mL methylprednisolone (depomedrol 40 mg) plus 1 mL of lignocaine 2%. The injection procedure was carried out about 1.5 fingerbreadths below the posterolateral corner of the acromion, without additional local anaesthesia. The needle was skillfully manoeuvred along the superior border of the rotator cuff into the subacromial space. If the needle tip contacted the undersurface of the acromion, slight withdrawal of the needle allowed smooth administration of the medication. Postinjection care instructions were identical for both groups.

### Outcomes

The primary outcome was the change in active shoulder forward flexion (degrees) at 3 months after injection. The secondary outcomes included changes in shoulder abduction, internal and external rotation, shoulder abduction strength (Medical Research Council scale) and patient‐reported outcome measures (SST and OSS). All outcomes were assessed at baseline and at 3‐month follow‐up by the same blinded orthopaedic assessor using standardised protocols. The assessor was different from the specialist who performed the injections.

### Clinical assessment

The clinical and functional parameters, including shoulder ROM, SST and OSS, were evaluated at baseline and 3 months after Ketorolac or CS injection. Active shoulder ROM was evaluated at baseline and at the 3‐month follow‐up by a blinded orthopaedic assessor using a standard universal goniometer. All measurements were performed with the patient seated comfortably in an upright position, with the trunk unsupported and the feet resting flat on the floor to minimise compensatory movements. The same standardised assessment protocol was applied consistently at both time points for all participants. Active forward flexion and abduction were measured as the maximum voluntary elevation of the arm in the sagittal and frontal planes, respectively. Patients were encouraged to move through their full comfortable range without assistance, and the terminal position was recorded in degrees. External rotation was assessed with the arm resting at the side of the body, the elbow flexed to 90° and the forearm in a neutral position. Patients were asked to rotate the forearm outward while keeping the elbow close to the trunk. This position was selected to enhance patient comfort and reproducibility in individuals with painful shoulder conditions. Internal rotation was measured with the shoulder positioned at 45° of forward flexion and the elbow flexed to 90°. From this position, patients actively rotated the forearm inward to the maximum comfortable range. This approach was chosen to allow consistent assessment of internal rotation while reducing pain‐related movement restriction commonly observed in SAIS. Each movement was measured once following a brief familiarisation trial, and all values were recorded in degrees. To ensure consistency, the same assessor, goniometer and measurement positions were used for all evaluations throughout the study. Muscle strength for shoulder abduction was qualitatively evaluated at baseline and 3 months after injection. This assessment was carried out using the Medical Research Council Manual Muscle Testing Scale, or the MRC scale, with the shoulder positioned at 45° abduction, the elbow at 90° flexion and the arm internally rotated without stabilising the torso. The MRC scale assesses muscle strength on a scale from 0 to 5: Grade 0 indicates no muscle activity or paralysis; Grade 1 shows visible muscle contraction without limb movement; Grade 2 involves limb movement through the full range but not against gravity; Grade 3 demonstrates limb movement against gravity but not with resistance; Grade 4 signifies movement against some resistance through the full range; and Grade 5 represents full movement and range against resistance, reflecting normal strength [18]. The SST consists of 12 shoulder‐specific questions to which the patient answers ‘yes’ or ‘no’. The questions ask about strength, function and ROM. 0 is considered the worst score, with 12 being the best score [1, 10]. The OSS contains 12 items, each with five answers ranging from 1 (best/fewest symptoms) to 5 (worst/most severe symptoms), which are awarded based on the patient's symptoms. The total score yields a maximum of 60, with higher scores indicating a greater degree of disability [1, 21].

### Concomitant physiotherapy

All participants received identical post‐injection instructions and were referred to a standardised physiotherapy program as part of routine care for SAIS. This program was consistently implemented across both groups without any treatment‐based modifications. The physiotherapy focused on shoulder mobility and strength, including ROM exercises, rotator cuff and scapular stabiliser strengthening and stretching routines. Participants were instructed to follow the prescribed program throughout the study. During follow‐up, no additional injections or group‐specific rehabilitation interventions were permitted.

### Adverse events

All participants were monitored for adverse events throughout the study. Adverse events were predefined as any local or systemic complication related to the injection, such as post‐injection pain, infection, skin changes, neurovascular symptoms or systemic reactions. Participants were specifically asked about adverse events at follow‐up visit, and none necessitated medical intervention or caused them to withdraw from the trial.

### Handling of missing data

Analysis followed the intention‐to‐treat principle. Participants lost to follow‐up were excluded only from analyses needing outcome data at 3 months. Due to the low attrition rate of two participants, no imputation methods were used. Baseline characteristics of participants lost to follow‐up were similar to those of the study completers.

### Statistical analysis

Statistical analyses were performed using SPSS ver. 27.0 (SPSS Inc., Chicago, IL, USA). Continuous variables were expressed as mean ± standard deviation (SD). Categorical data were expressed as percentage and frequency. Continuous numeric variables were assessed for the normality of their distribution using the Kolmogorov–Smirnov test. Given the normal distribution of the data, continuous variables are presented as mean ± SD, and between‐group comparisons are performed using Student's *t‐*tests. The significance level was set at *p* < 0.05 for all analyses. Effect sizes are reported as Cohen's *d*. Positive values indicate a preference for CS; negative values indicate a preference for ketorolac. The noninferiority margin (Δ) was set at −10° for all ROM measures. This margin accounts for about 50% or less of the minimal clinically important difference reported for shoulder ROM in patients with SAIS and exceeds the measurement error associated with goniometry. Consequently, a loss of less than 10° was deemed clinically acceptable [6]. Noninferiority was assessed using baseline‐adjusted analyses of covariance (ANCOVA), with the 3‐month ROM as the dependent variable, treatment group as the fixed factor and baseline ROM as a covariate. Adjusted between‐group differences were reported as ketorolac minus CS, with corresponding two‐sided 95% confidence intervals (95% CI). Ketorolac was considered non‐inferior if the lower bound of the confidence interval exceeded the predefined margin of −10°.

## RESULTS

### General characteristics

**Figure 1:**
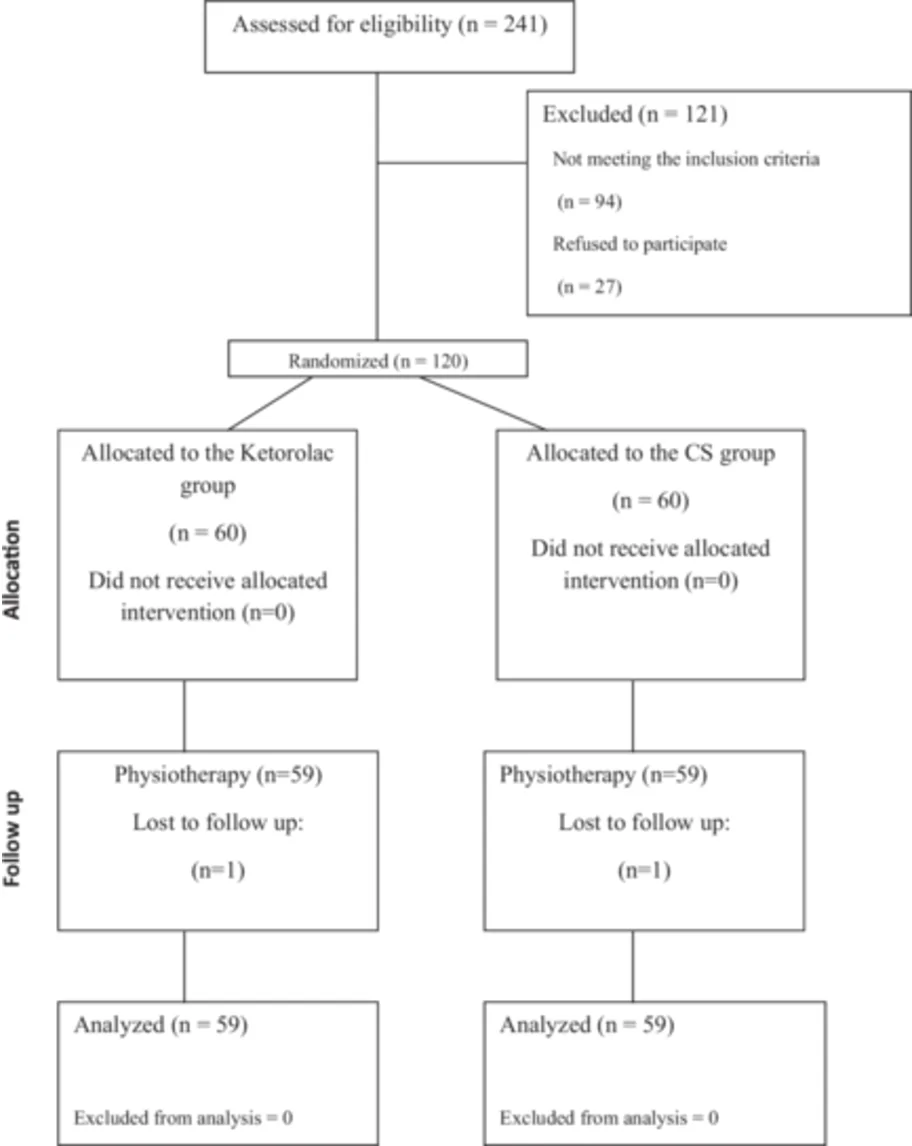
CONSORT flow diagram showing participants inclusion, exclusion and grouping.

Among the 241 individuals who visited our clinic between March 2022 and March 2023, 147 patients were eligible for enrolment in the study. Of these, 120 provided informed consent and were randomised into groups at an allocation ratio of 1:1 (60 in the Ketorolac group and 60 in the CS group). Two participants were lost to follow‐up, resulting in 118 patients (59 in each group) being included in the final analysis. Figure 1 illustrates the CONSORT flow diagram of patient inclusion and grouping. Baseline demographic and clinical characteristics were comparable between the two groups (Table 1). The mean age was 59.8 ± 11.3 years in the CS group and 55.9 ± 13.7 years in the Ketorolac group (*p* = 0.09). Sex distribution was identical in both groups (49% male, 51% female). There were no statistically significant differences in body mass index (*p* = 0.42), symptom duration (*p* = 0.20), hand dominance (*p* = 0.36), smoking status (*p* = 0.71) or comorbidities, including diabetes mellitus (*p *= 0.31) and hypertension (*p* = 1.0). Only a borderline difference was noted in the prevalence of ischaemic heart disease (15% in CS vs. 3% in ketorolac; *p* = 0.053). Overall, randomisation achieved well‐balanced baseline characteristics.

**Table 1:** Baseline demographic characteristics of participants.

Groups			
Parameters	CS group *N* = 59	Ketorlac group *N* = 59	*p* value
Sex (male: female)	29 (49%): 30 (51%)	29 (49%): 30 (51%)	1.0
Age (year)	59.8 ± 11.3	55.9 ± 13.7	0.09
Body mass index (BMI)	26.2 ± 4.1	25.6 ± 3.9	0.42
Symptom duration (months)	16.8 ± 14.8	13.9 ± 9.2	0.20
Lesion laterality (Left: Right)	32 (54%): 27 (46%)	23 (39%): 36 (61%)	0.09
Dominant hand (Left: Right)	31 (53%): 28 (48%)	26 (44%): 33 (56%)	0.36
Smoking	27 (46%)	29 (49%)	0.71
Diabetes mellitus	11 (19%)	7 (12%)	0.31
Hypertension	7 (12%)	6 (10%)	1.0
Ischaemic heart disease	9 (15%)	2 (3%)	0.053
Hyperlipidemia	2 (3%)	2 (3%)	1.0

Abbreviation: CS, corticosteroid.

### Clinical parameters

**Figure 2:**
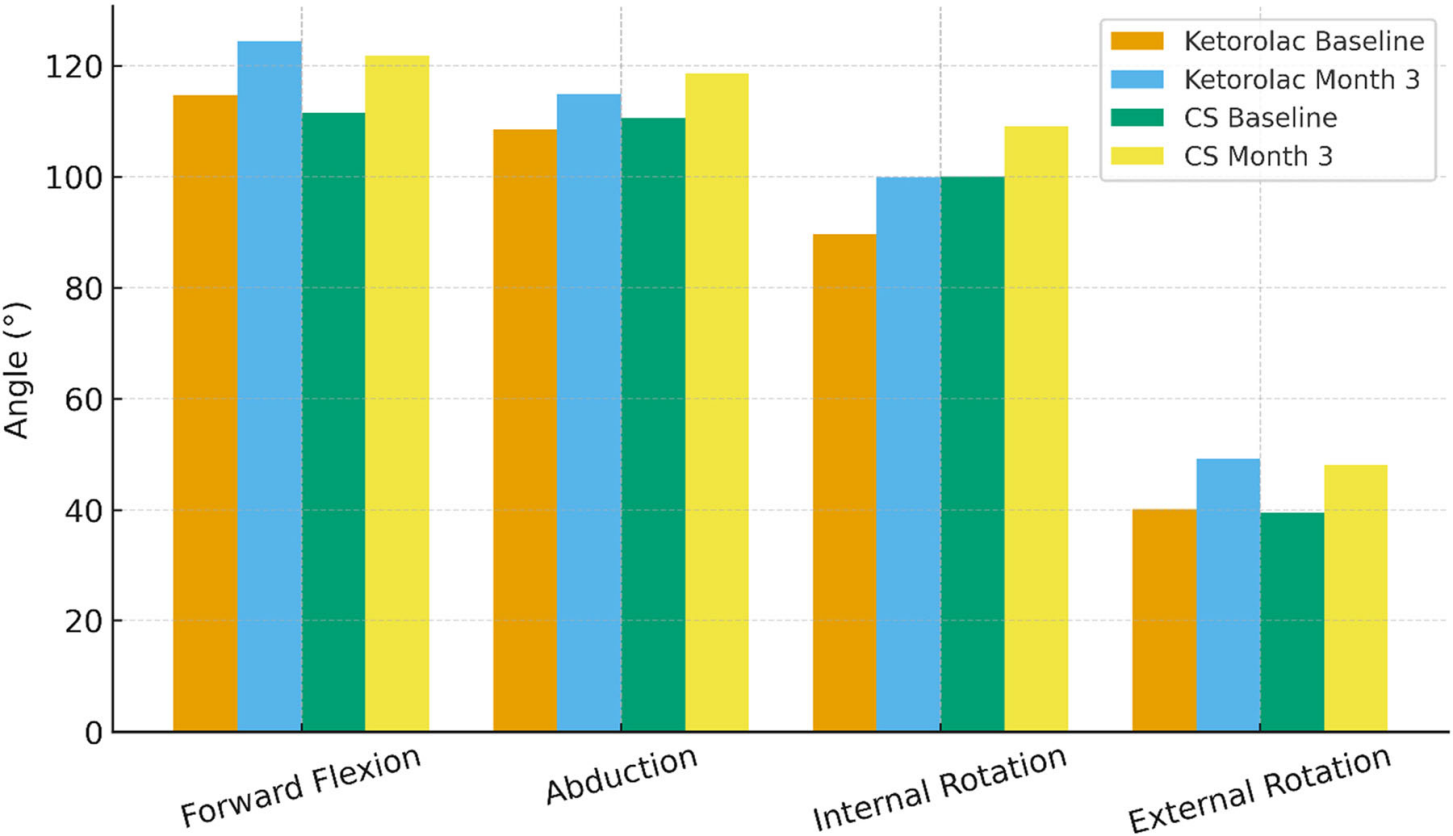
Shoulder ROM angles comparison. CS, corticosteroid; ROM, range of motion.

All parameters showed statistically significant differences compared to baseline in both groups (*p* < 0.001). There was no statistically significant difference in forward flexion, abduction, external rotation range or abduction strength at baseline and the 3‐month follow‐up between the two groups (*p* > 0.05). Internal rotation range was significantly higher in the CS group at both baseline and the 3‐month follow‐up (*p* < 0.05). Additionally, regarding the change over the 3 months, there was no statistically significant difference between the two groups (*p* > 0.05) (Table 2 and Figure 2).

**Table 2:** Clinical outcomes.

Outcome	Baseline	Month 3	Change
**Forward flexion (◦)**			
Ketorolac (*N* = 59)	114.6 ± 32.3 (95% CI: 106.1–123.0)	124.4 ± 30.1 (95% CI: 116.6–132.3)	9.8 ± 12.5 (95% CI: 6.6–13.1)
CS (*N* = 59)	111.5 ± 35.3 (95% CI: 102.3–120.7)	121.7 ± 33.0 (95% CI: 113.1–130.3)	10.2 ± 12.8 (95% CI: 6.8–13.5)
*p*‐Value	0.63	0.64	0.89
Effect size (Cohen's *d*)	−0.09 (95% CI: −0.45 to 0.27)	−0.09 (95% CI: −0.45 to 0.28)	0.03 (95% CI: −0.33 to 0.39)
**Abduction (◦)**			
Ketorolac (*N* = 59)	108.5 ± 31.2 (95% CI: 100.3–116.6)	114.8 ± 33.5 (95% CI: 106.1–123.6)	6.4 ± 11.3 (95% CI: 3.4–9.3)
CS (*N* = 59)	110.5 ± 25.6 (95% CI: 103.8–117.2)	118.5 ± 26.4 (95% CI: 111.9–125.7)	8.3 ± 12.6 (95% CI: 5.0–11.6)
*p*‐Value	0.70	0.48	0.38
Effect size (Cohen's *d*)	0.07 (95% CI: −0.29 to 0.43)	0.13 (95% CI: −0.23 to 0.49)	0.16 (95% CI: −0.20 to 0.52)
**Internal rotation (◦)**			
Ketorolac (*N* = 59)	89.7 ± 27.2 (95% CI: 82.6–96.8)	99.8 ± 24.8 (95% CI: 93.4–106.3)	10.2 ± 10.9 (95% CI: 7.3–13.0)
CS (*N* = 59)	100.0 ± 21.6 (95% CI: 94.4–105.6)	109.0 ± 22.7 (95% CI: 103.1–114.9)	9.0 ± 18.0 (95% CI: 4.3–13.7)
*p*‐Value	0.02	0.04	0.67
Effect size (Cohen's *d*)	0.42 (95% CI: 0.06–0.79)	0.39 (95% CI: 0.02–0.75)	−0.08 (95% CI: −0.44–0.28)
**External rotation (◦)**			
Ketorolac (*N* = 59)	40.2 ± 18.1 (95% CI: 35.4–44.9)	49.2 ± 15.5 (95% CI: 45.1–53.2)	9.0 ± 11.2 (95% CI: 6.1–11.9)
CS (*N* = 59)	39.5 ± 13.8 (95% CI: 35.9–43.1)	48.1 ± 12.9 (95% CI: 44.8–51.5)	8.6 ± 11.2 (95% CI: 5.7–11.6)
*p*‐Value	0.82	0.70	0.87
Effect size (Cohen's *d*)	−0.04 (95% CI: −0.40 to 0.32)	−0.07 (95% CI: −0.43 to 0.29)	−0.03 (95% CI: −0.39 to 0.33)
**Abduction strength (grade (2 or 3)/4/5)**			
Ketorolac (*N* = 59)	31/26/2	18/32/9	
CS (*N* = 59)	23/32/4	17/31/11	
*p*‐Value	0.29	0.89	

Abbreviations: CI, confidence intervals; CS, corticosteroid.

### Patient‐reported outcome measures (PROMs)

**Figure 3:**
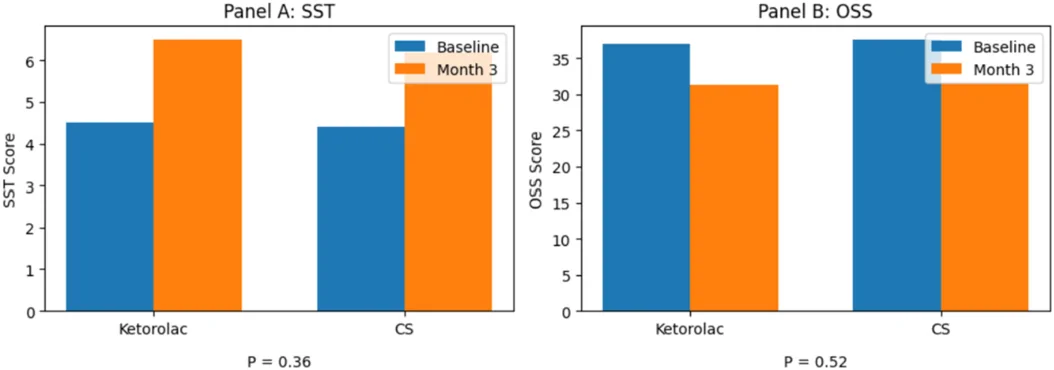
PROMs comparison. OSS, Oxford shoulder score; PROMs, patient‐reported outcome measure; SST, simple shoulder test.

The SST and OSS showed statistically significant differences from baseline in both groups (*p* < 0.001). SST increased from 4.5 ± 3.1 to 6.5 ± 3.2 in the Ketorolac group and from 4.4 ± 2.9 to 6.2 ± 3.2 in the CS group, and OSS improved (lower scores indicate better outcomes) from 37.0 ± 6.9 to 31.3 ± 6.0 in the Ketorolac group and from 37.6 ± 6.4 to 31.4 ± 5.2 in the CS group. There was no statistically significant difference between the two groups in these scores at baseline and at the 3‐month follow‐up (*p* > 0.05). Notably, regarding the amount of change over the 3 months, there was no statistically significant difference between the two groups (*p* > 0.05) (Table 3 and Figure 3). Overall, both treatments yielded clinically and statistically significant within‐group improvement in pain and shoulder function; however, no superiority of one intervention over the other was observed.

**Table 3:** Patient‐reported outcome measures.

Outcome	Baseline	Month 3	Change
**SST**			
Ketorolac (*N* = 59)	4.5 ± 3.1	6.5 ± 3.2	2.1 ± 1.7
CS (*N* = 59)	4.4 ± 2.9	6.2 ± 3.2	1.8 ± 1.5
*p*‐value	0.83	0.51	0.36
**OSS**			
Ketorolac (*N* = 59)	37.0 ± 6.9	31.3 ± 6.0	−5.8 ± 3.1
CS (*N* = 59)	37.6 ± 6.4	31.4 ± 5.2	−6.1 ± 3.2
*p*‐value	0.67	0.88	0.52

Abbreviations: CS, corticosteroid; OSS, Oxford shoulder score; SST, simple shoulder test.

### Non‐inferiority analysis of primary outcome

In the baseline‐adjusted analysis, forward flexion at 3 months did not differ meaningfully between the ketorolac and CS groups. The adjusted mean difference (ketorolac minus CS) was 0.07° (95% CI: −4.40° to 4.26°). The lower bound of the confidence interval exceeded the Δ of −10°, thereby demonstrating noninferiority of ketorolac with respect to forward flexion. Baseline forward flexion was a significant predictor of follow‐up motion (*p* < 0.001), supporting the validity of the ANCOVA model. No clinically relevant between‐group differences were observed (Table 4).

**Table 4:** Non‐inferiority analysis of primary outcome at 3‐month follow‐up (baseline‐adjusted ANCOVA).

Outcome	Ketorolac (adjusted mean ± SE)	CS (adjusted mean ± SE)	Adjusted mean difference (Ketorolac—CS)	95% CI	Non‐inferiority margin (Δ)	Non‐inferior
**Forward flexion**	123.09 ± 1.55	123.02 ± 1.55	0.07	−4.40 to 4.26	−10	Yes

Abbreviations: ANCOVA, analyses of covariance; CI, confidence intervals; CS, corticosteroid; SE, standard error.

### Adverse events

During the 3‐month follow‐up, neither treatment group reported any injection‐related adverse events. Specifically, there were no instances of infection, ongoing post‐injection pain, neurovascular issues, skin or subcutaneous changes, tendon rupture or systemic side effects. Additionally, no participants needed further medical care or withdrew from the study due to complications from the intervention.

## DISCUSSION

The main finding of this randomised controlled trial is that a single subacromial ketorolac injection is noninferior to a CS injection in improving active shoulder forward flexion at 3 months in patients with SAIS. Both treatments produced statistically and clinically significant improvements in shoulder ROM, muscle strength and patient‐reported outcomes, with no meaningful differences between groups. These results support our a priori hypothesis that ketorolac provides clinical improvement comparable to CSs.

The noninferiority analysis of the primary outcome supports this conclusion. The baseline‐adjusted mean difference in forward flexion between groups was minimal, and the lower bound of the 95% confidence interval remained well above the prespecified non‐inferiority margin of −10°, confirming clinically comparable effectiveness. Similarly, both groups demonstrated significant within‐group improvements in all secondary outcomes, including shoulder ROM, abduction strength and SST and OSS scores, with no significant between‐group differences over time. Although internal rotation was greater in the CS group at baseline and follow‐up, this pre‐existing difference limits causal interpretation, and no clear treatment advantage was observed.

Our results align with prior studies showing that both CS and Ketorolac injections provide meaningful pain relief and functional improvement in shoulder impingement and rotator cuff tendinopathy. Taheri et al. conducted the first RCT on ketorolac application for shoulder impingement syndrome in the Iranian population, with a 3‐month follow‐up of the Ketorolac and CS group, with a smaller sample size, each group containing 20 patients. They found that in 1 and 3 months, both treatment groups showed an increased ROM (according to Constant's score) and decreased pain (based on visual analog scale [VAS]). The difference between the groups was not statistically significant (*p* > 0.05). Ketorolac produced similar results to CS in most clinical aspects among patients with shoulder impingement syndrome [22]. Likewise, in a study by Goyal et al., the effectiveness of subacromial ketorolac and CS injections in treating SAIS was compared with a final sample size of 67 patients. Both treatments significantly reduced pain (according to VAS) and improved shoulder function (according to the shoulder pain and disability score [SPAD] and ROM) over 3 months, with no statistically significant difference between groups [11]. Min et al. compared the effectiveness of a single subacromial injection of Ketorolac versus triamcinolone in 32 patients with impingement syndrome. They found that, at 4 weeks, both injections resulted in increased ROM and decreased pain. Ketorolac injections demonstrated superior efficacy compared to triamcinolone, regarding improvements in University of California at Los Angeles (UCLA) shoulder scores [17]. The findings suggest ketorolac may be a safe and effective alternative to CSs, especially when steroid use is contraindicated.

Intraarticular Ketorolac is regarded as a safe alternative to intra‐articular CSs according to the current literature. Animal model studies involving knee intra‐articular administration of NSAIDs have shown no damaging effects on articular ligaments or cartilage, nor any adverse impact on kinematic function [14]. In vitro studies with bovine tissue suggest that ketorolac not only does not harm cartilage but may also offer protective benefits by inhibiting cytokines such as interleukin‐1, a known trigger for cartilage damage during inflammation [3, 14, 20]. Jianda et al. evaluated the pain‐relief benefits of Ketorolac compared to CSs when used alongside sodium hyaluronate injections for knee osteoarthritis. Among 42 patients who received five weekly injections of Ketorolac, only three participants had mild, localised pain after injections, lasting approximately 1–3 days. These pain episodes were mild, self‐limited and resolved on their own without needing further treatment [27].

This study has some limitations. First, the follow‐up period was only 3 months. While this duration is enough to observe short‐term responses to the subacromial injection, it cannot assess long‐term effectiveness, lasting symptom relief or late‐onset adverse effects, especially those that might appear with repeated treatments. Therefore, the results should be viewed as reflecting only short‐term outcomes. Longer‐term results, such as imaging assessments and structural improvements visible on radiology, were not examined. The diagnosis of SAIS was based on clinical assessment. In some cases, CS injections tend to be ineffective when administered several weeks after the onset of the pathology [26], which could not be evaluated in our study design. In addition, since this was a clinical trial, a few participants missed follow‐up appointments and were lost to follow‐up during the study.

## CONCLUSION

Both subacromial ketorolac and CS injections led to significant short‐term improvements in shoulder ROM and function in patients with SAIS. No meaningful differences were found between the groups at 3 months. Ketorolac demonstrated similar effectiveness to CSs for the primary outcome within the established non‐inferiority margin. No adverse events related to the injections occurred during follow‐up. However, due to the short duration, conclusions about long‐term efficacy or safety cannot be made. Larger studies with longer follow‐up are needed.

## AUTHOR CONTRIBUTIONS

All authors participated in the conception and design of the study. **Omid Salkhori**: Conceptualisation; data collection; data analysis; investigation; drafting of the manuscript. **Mahdi Sahebi**: Data acquisition; methodology; statistical analysis; drafting of the manuscript. **Mohammad Reza Guity**: Supervision; project administration; critical revision of the manuscript for important intellectual content. **Saman Ghiasi**: Methodology; visualisation; validation of data accuracy. **Nima Bagheri**: Study conception and design; overall supervision; interpretation of data; final edit of the manuscript. All authors read and approved the final version of the manuscript and agree to be accountable for all aspects of the work.

## CONFLICT OF INTEREST STATEMENT

The authors declare no conflicts of interest.

## ETHICS STATEMENT

Yes, the IRB code was obtained from the Institutional Review Board of the Tehran University of Medical Sciences (IR.TUMS.IKHC.REC.1401.315). Informed consent was obtained from all patients.

## Data Availability

The data analysed during the current study are not publicly available due to patient confidentiality and institutional restrictions, but are available from the corresponding author on reasonable request. All data were collected at the Imam Khomeini Hospital Complex, Tehran University of Medical Sciences, under the approved ethical protocol (IR.TUMS.IKHC.REC.1401.315). The study was registered with the Iranian Registry of Clinical Trials (IRCT20230625058584N1).
